# A clinical 3D/4D CBCT‐based treatment dose monitoring system

**DOI:** 10.1002/acm2.12474

**Published:** 2018-10-10

**Authors:** An Qin, David Gersten, Jian Liang, Qiang Liu, Inga Grill, Thomas Guerrero, Craig Stevens, Di Yan

**Affiliations:** ^1^ Department of Radiation Oncology Beaumont Health System Royal Oak MI USA

**Keywords:** 4D‐CBCT, adaptive radiotherapy, CBCT, IGRT, treatment dose

## Abstract

To monitor delivered dose and trigger plan adaptation when deviation becomes unacceptable, a clinical treatment dose (Tx‐Dose) reconstruction system based on three‐dimensional (3D)/four‐dimensional (4D)‐cone beam computed tomograpy (CBCT) images was developed and evaluated on various treatment sites, particularly for lung cancer patient treated by stereotactic body radiation therapy (SBRT). This system integrates with our treatment planning system (TPS), Linacs recording and verification system (R&V), and CBCT imaging system, consisting of three modules: Treatment Schedule Monitoring module (TSM), pseudo‐CT Generating module (PCG), and Treatment Dose Reconstruction/evaluation module (TDR). TSM watches the treatment progress in the R&V system and triggers the PCG module when new CBCT is available. PCG retrieves the CBCTs and performs planning CT to CBCT deformable registration (DIR) to generate pseudo‐CT. The 4D‐CBCT images are taken for target localization and correction in lung cancer patient before treatment. To take full advantage of the valuable information carried by 4D‐CBCT, a novel phase‐matching DIR scheme was developed to generate 4D pseudo‐CT images for 4D dose reconstruction. Finally, TDR module creates TPS scripts to automate Tx‐Dose calculation on the pseudo‐CT images. Both initial quantitative commissioning and patient‐specific qualitative quality assurance of the DIR tool were utilized to ensure the DIR quality. The treatment doses of ten patients (six SBRT‐lung, two head and neck (HN), one breast and one prostate cancer patients) were retrospectively constructed and evaluated. The target registration error (mean ± STD: 1.05 ± 1.13 mm) of the DIR tool is comparable to the interobserver uncertainty (0.88 ± 1.31 mm) evaluated by a publically available lung‐landmarks dataset. For lung SBRT patients, the D_99_ of the final cumulative Tx‐Dose of GTV is 93.8 ± 5.5% (83.7–100.1%) of the originally planned D_99_. CTV D_99_ decreases by 3% and mean ipsilateral parotid dose increases by 11.5% for one of the two HN patients. In conclusion, we have demonstrated the feasibility and effectiveness of a treatment dose verification system in our clinical setting.

## 1 INTRODUCTION

Treatment delivered dose in patient could vary from the planned one due to patient setup, target motion, and anatomic variation.^1^, ^2^ For lung cancer treatment, the percentage of ITV volume changes of a cohort of 40 Stereotactic Body Radiation Therapy (SBRT) lung patients were reported to range from −59.6% to 13.0% (−21.0 ± 21.4%) at the end of treatment.^3^ The anatomic change caused by atelectasis, pleural effusion, and pneumonia could also significantly affect the dose distribution.^4^, ^5^ In addition, the breathing pattern variations during treatment in magnitude, period, and mean position could negatively impact the delivered dose.^6^ For head and neck cancer patient, Vasquez Osorio et al. reported that the primary tumor shrunk by 25 ± 15% and the ipsilateral parotid grand by (20 ± 10%).^7^ For radiotherapy in the pelvic and abdominal region, the organ filling and deformation, which may not be fully correctable by couch shifting, could lead to significant treatment dose deterioration of critical organs.^8^, ^9^, ^10^, ^11^, ^12^ As a result, treatment dose assessment is a useful technique to monitor delivery accuracy for patients who may undergo notable tumor regression/progression or anatomy/motion variation.

A clinical treatment dose monitoring system can serve as an important infrastructure to support the radiotherapy treatment quality evaluation, adaptive radiation therapy decision‐making, and treatment outcome modelling.^13^, ^14^ For example, the reconstructed daily treatment dose can be utilized to monitor the target coverage and organ at risk (OAR) sparing during the delivery. The cumulative treatment dose from previously delivered fractions can be used to support plan adaptation decisions.^15^ Among all the factors, patient anatomic variation during radiotherapy is considered to be the number one source of uncertainty for radiobiology modeling.^16^ Therefore, the reconstruction of cumulative treatment dose based on CBCT could became a prerequisite for accurate treatment outcome modeling.^2^, ^17^

Numerous studies have demonstrated the feasibility of using CBCT to construct treatment dose for photon^18^, ^19^, ^20^, ^21^, ^22^, ^23^ and recently for proton radiotherapy^24^, ^25^ on various treatment sites. However, few such systems actually are running in real clinical routine due to various practical reasons. Three major obstacles could be the limited interoperability among different software systems in a specific clinical setting, the extra clinical workload for an already busy clinic and the concern of deformable image registration (DIR) uncertainty behind dose accumulation.^26^, ^27^, ^28^ Ideally, such a system should integrate seamlessly with the existing treatment planning system, recording and verify system, and onboard imaging system. All the necessary information such as treatment plan, delivery schedule, and CBCT image should be retrieved automatically. Also, the clinical workflow should be as intuitive and automate as possible to minimize the extra clinical workload. Furthermore, as emphasized in the recently published AAPM Task Group report 132 (TG 132), a convenient patient‐specific quality assurance (QA) of DIR between CT and CBCT is essential to ensure the quality of pseudo‐CT creation and treatment dose warping^29^.

Early stage non‐small cell lung tumor could be difficult to identify on 3D‐CBCT due to inferior image quality and motion artifact.^30^, ^31^ The 4D‐CBCT technique has been widely adopted for daily image‐guided alignment of moving targets.^32^ Daily breathing pattern variation and density distribution change of surrounding tissue, as manifested on 4D‐CBCT, carry critical information of the treatment day for dose reconstruction. For example, the intrafraction variation of mean target position was reported to be 2.3 ± 2.1 mm based on a total of 126 patients who underwent 659 fractions of lung SBRT,^33^ and 7.2% of the fractions had mean position change larger than 5 mm. Treatment dose monitoring, taking full advantage of 4D‐CBCT, will provide additional support and confidence for the highly conformal and hypofractionated techniques like SBRT lung cancer treatment.^34^ A complete treatment dose reconstruction, either online or offline based on 4D‐CBCT, will give clinicians the opportunity to review the potential dosimetric impact of the anatomical and motion variations.

The purpose of this work was to develop and evaluate the feasibility of a semiautomatic 3D/4D‐CBCT‐based treatment dose monitoring system in our clinical setting for routine treatment dose tracking.

## 2 MATERIAL AND METHODS

### 2.A System architecture

**Figure 1:**
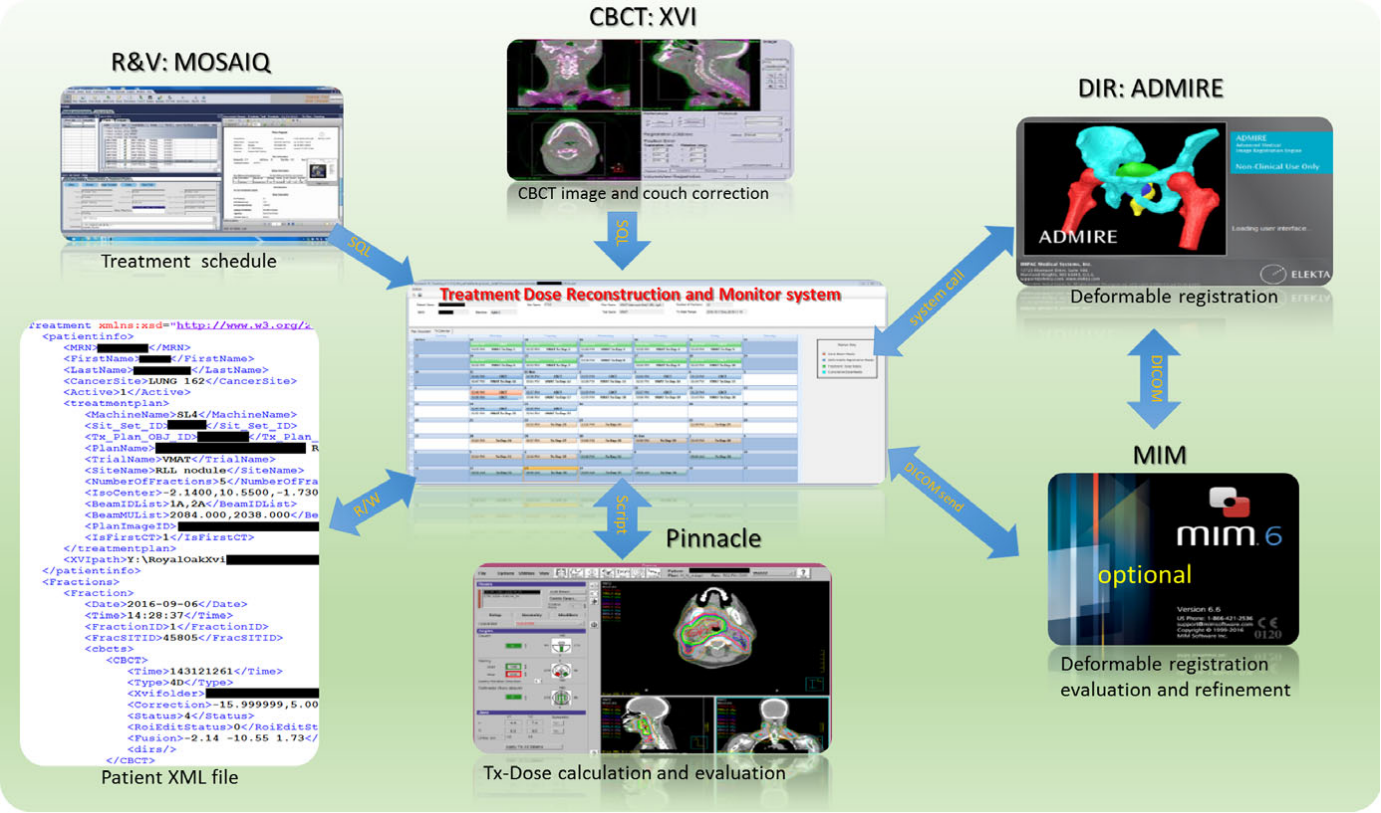
The integration of the dose monitoring system with our TPS, R&V, and CBCT imaging system.

The system was interfaced with Pinnacle Treatment Planning System (TPS, version 14, Philips Medical Systems, Madison, WI), MOSAIQ Record and Verify system (R&V, version 2.64, Elekta, Stockholm, Sweden), and CBCT imaging system (XVI, version 5.0, Elekta) to reconstruct daily delivered dose in patients. This system included three modules: Treatment Schedule Monitoring module (TSM), pseudo‐CT Generating Module (PCG), and Treatment Dose Reconstruction/evaluation module (TDR). An illustration of the interactions of the system with other clinical software/systems is depicted in Fig. 1.

#### 2.A.1 TSM

When a new patient is added to the tracking list, the system queries the patient's treatment schedule from the MOSAIQ database and generates an Extensible Markup Language (XML) file which describes the treatment delivery process, including general patient and treatment information, as well as the scheduled fractions. TSM refreshes the patient XML file automatically to synchronize with the latest treatment progress. When a fraction is delivered and CBCTs are ready, PCG module is called to generate pseudo‐CT.

#### 2.A.2 PCG

PCG module automatically retrieves CBCT images and couch corrections from CBCT imaging system database (XVI). A research version of a commercial DIR tool (ADMIRE 2.0, Elekta Inc.) is utilized for the DIR between CT and CBCT. Briefly, the intrapatient algorithm of ADMIRE performs a block‐wise nonlinear registration to get a robust initial alignment, followed by a dense local correlation coefficient (LCC)‐based deformable registration to get the final deformable vector field (DVF). This tool has been reported and evaluated in several international challenges of head and neck and lung patients DIR with high‐ranking results.^35^, ^36^, ^37^, ^38^ It was also comprehensively evaluated in our institution for Head and Neck cancer patient with expert‐delineated contours as ground truth, including seven OARs (brain stem, cord, L/R parotids, L/R submandibular gland, and mandible).^39^ The CT–CT intrapatient propagation achieved Dice similarity coefficient (DICE) greater than 0.85 and (Mean surface distance) MSD smaller than 1.2 mm. The DICE and MSD of CT–CBCT propagation were very close to the CT–CT results, decreasing only by 0.03 and 0.2 mm respectively.

**Figure 2:**
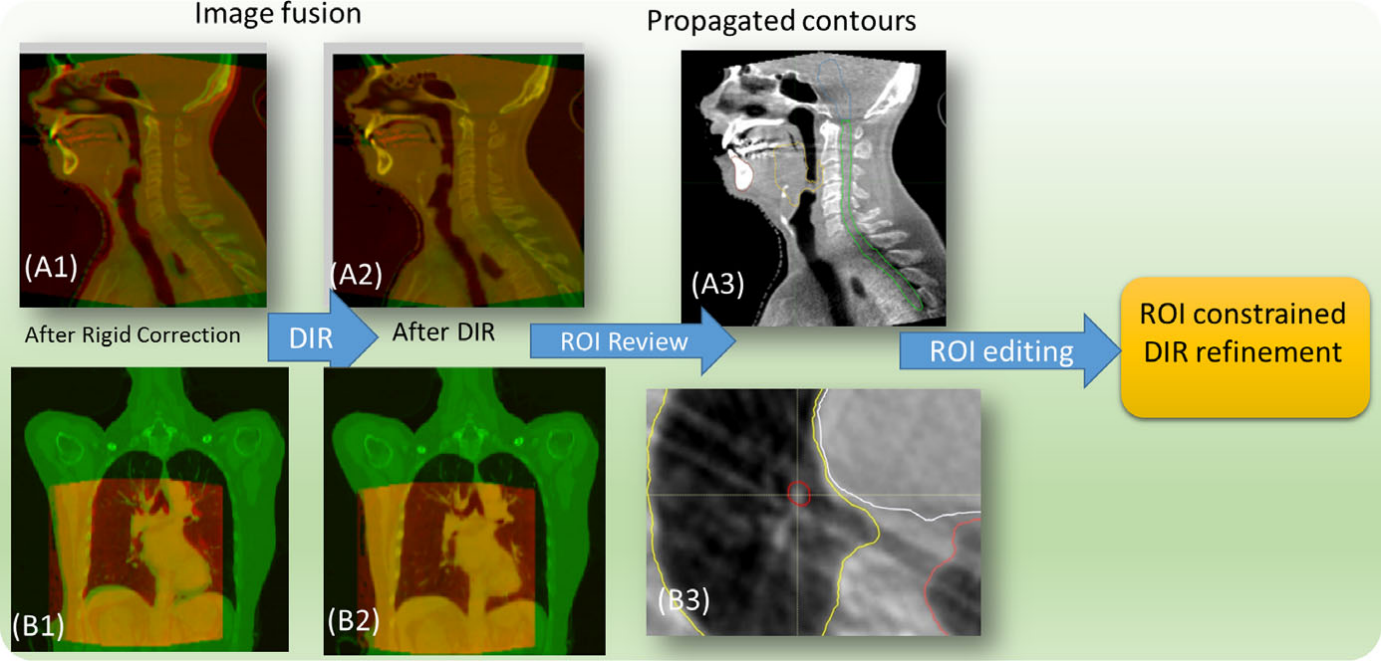
Qualitative inspection of CBCT–CT deformable registration by fusion images and propagated contours: CT in green and CBCT in red, A1–A3 head and neck patient, B1–B3 phase‐matching lung CBCT.

The pretreatment planning CT is set as the reference image and the CBCT as the moving image during the DIR. Since the field of view (FOV) of the CBCT is usually smaller, the CT is cropped to match the FOV of the CBCT to avoid unrealistic DIR distortion across the boundary. The DVF on small FOV of CBCT is expanded to the FOV of the CT by smoothing over the FOV boundary with a Gaussian filter, ensuring a smooth transition. Backward DVF from CBCT to CT coordinates is generated based on the forward one. The intensity overrides applied on the plan CT, for example for dental fill or mental artifact, are exported from TPS and used to override the original CT pixel. The resulting CT number is then mapped to the CBCT coordinate via the backward DVF to generate a pseudo‐CT. For each DIR, fusion images before and after DIR as well as the propagated region of interest (ROI) on the CBCT, are generated for patient‐specific DIR QA. The fusion images and the propagated ROIs on CBCT are shown for a head and neck patient in the top row and lung patient in bottom row, respectively in Fig. 2. On these fusion images, the CT in green was deformed toward the CBCT in red to generate the pseudo‐CT. If the DIR quality is not satisfactory, the propagated contour can be sent to MIM (MIM Software, Cleveland, OH) for modification. The modified contour can then be transferred back to guide the DIR algorithm for further DVF refinement (ROI‐constrained DIR), as shown in Fig. 1 as an optional step.

#### 2.A.3 TDR

This module takes care of the treatment dose recalculation. When a pseudo‐CT is ready, TDR is triggered to generate a script file that automates the following steps: loading of pseudo‐CT into TPS, dose recalculation and exportation of daily treatment dose. The forward DVF from the DIR is then used to warp/accumulate the dose to the planning CT for direct comparison with planned dose. If a patient undergoes one or more plan adaptations, the treatment dose will be warped to the nearest replan CT first and then warped to the pretreatment CT. The warped doses can then be loaded back to TPS for evaluation via a graphic user interface (GUI) that facilitates the selection of treatment date and dose type (daily/cumulative) as shown in Fig. 4.

### 2.B Phase‐matching DIR and treatment dose reconstruction for lung SBRT

To accurately reconstruct treatment dose for lung cancer patients, the daily target motion and density distribution variation from 4D‐CBCT have to be utilized. A novel phase‐matching DIR scheme was developed for deformable registration of 4D‐CT to 4D‐CBCT. First, to minimize the anatomic difference before DIR, the closest matching phases, based on diaphragm position, are identified automatically from the planning 4D‐CT and daily 4D‐CBCT. The paired phase images are then deformable registered with each other. The CT is cropped to match the FOV of CBCT as described in Section 2.A‐PCG. The CT number is then warped to corresponding CBCT to generate 4D‐pseudo‐CT. Fusion images of an example lung patient's matching phase are shown in Fig. 2B(1–2) before and after DIR, with CT in green and CBCT in red. The overlapping of the skin, diaphragm, and heart has been improved significantly after DIR.

**Figure 3:**
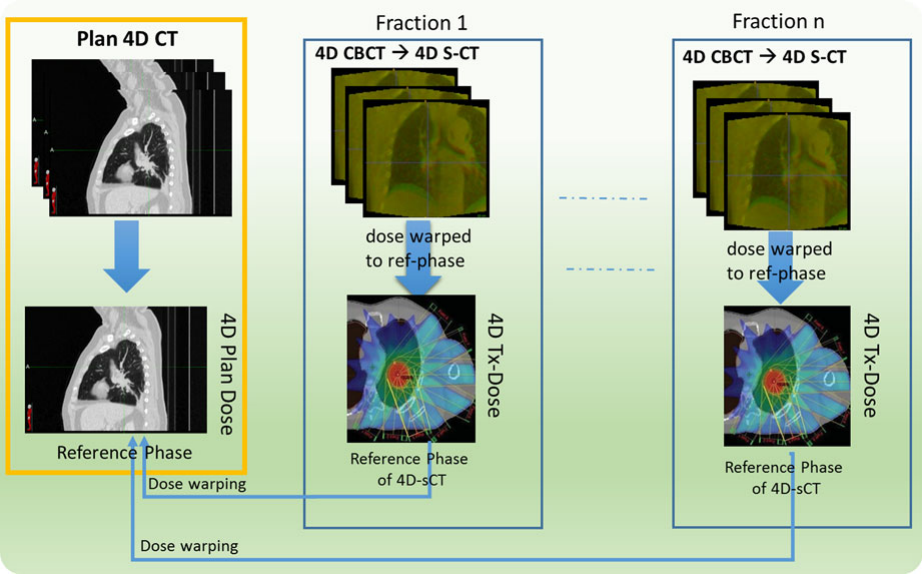
4D treatment dose reconstruction based on 4D‐CBCT for lung cancer patient.

A diagram illustrating the 4D‐CBCT–based treatment dose reconstruction is shown in Fig. 3. After the 4D‐pseudo‐CT is generated, the images are load into TPS to calculate doses on all ten phases. All the phase doses are then warped to the reference phase of 4D‐pseudo‐CT (End of inhale) via DIR. The 4D treatment dose (4D Tx‐Dose) is then warped from the coordinate of reference phase of 4D‐pseudo‐CT to that of the planning 4D‐CT for the convenience of evaluation. TDR module will generate a script to automate the 4D treatment dose reconstruction/warping. The reconstructed 4D Tx‐Dose should incorporate all the anatomic and breathing pattern variation as manifested on 4D‐CBCT.

### 2.C The clinical workflow

**Figure 4:**
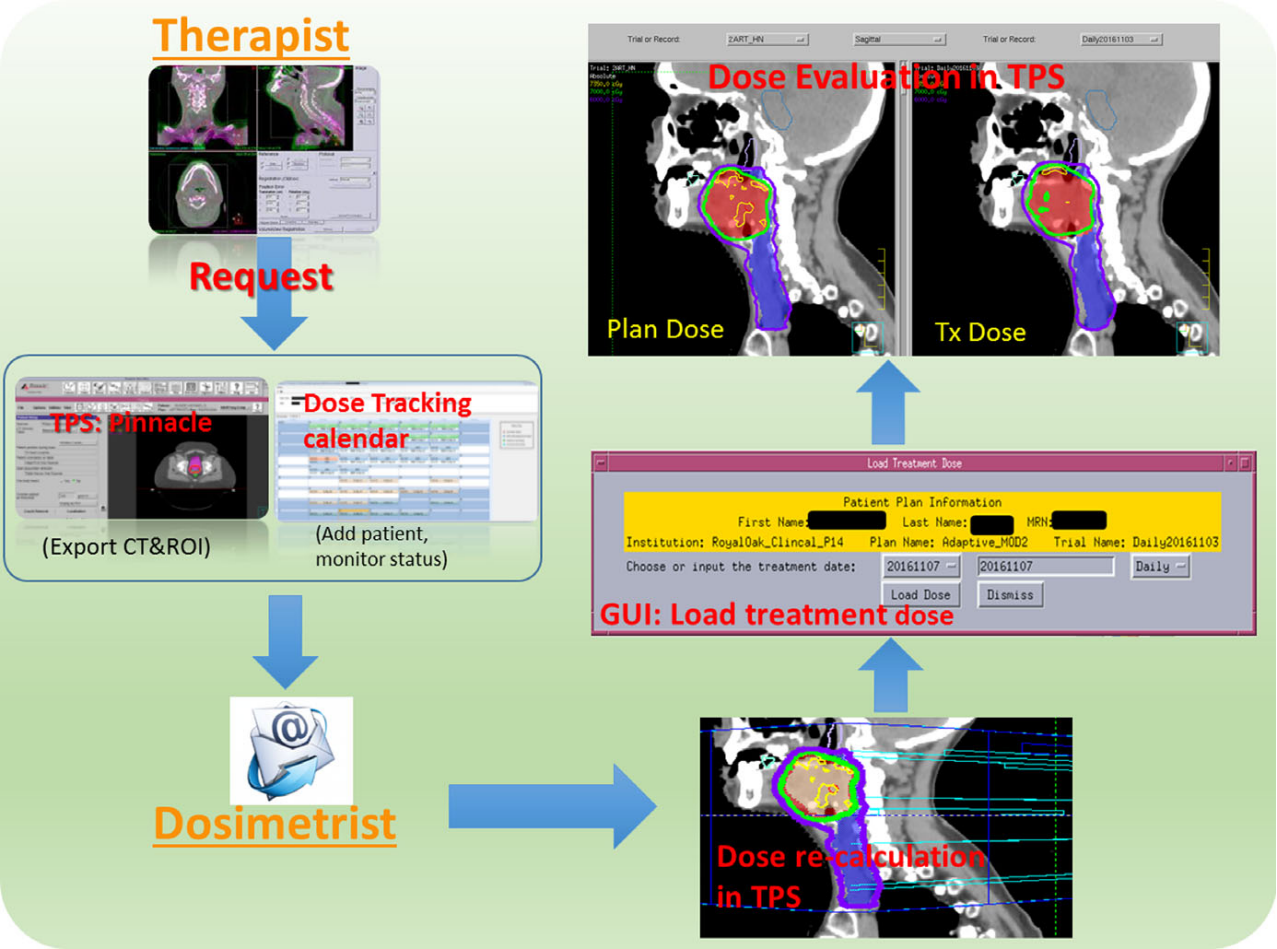
Clinical workflow of the treatment dose monitoring process.

The clinical workflow as illustrated in Fig. 4 was implemented for treatment dose monitoring: (a) Request: therapist, who performs daily CBCT‐based patient alignment, will request treatment dose reconstruction when significant anatomic/motion variation is observed during treatment. (b) Preparation: dosimetrist adds the patient to the watching list and runs a script in TPS to export all plan information necessary for pseudo‐CT generation (ROI, density override, couch position et al.). (c) Monitoring: the system automatically monitors the radiotherapy progress by interfacing with R&V system and performs treatment dose reconstructions. A calendar GUI (Fig. 1 center) was designed to visualize the progress with four successive statuses for each fraction [(a) CBCT ready, (b) pseudo‐CT ready, (c) Tx‐dose ready, (d) cumulative dose ready]. The request and preparation steps need human operation and the monitoring step is mostly automatic. When a treatment fraction is delivered (R&V database updated) and the CBCT is ready, the system will automatically start to perform deformable registration and generate a pseudo‐CT. TPS scripts are then generated to calculate treatment dose when the pseudo‐CT is ready. Finally, the treatment doses are warped into plan CT coordinate to get daily and cumulative Tx‐dose for evaluation. (d) Evaluation: Dosimetrist will receive email notification when Tx‐doses are ready and evaluate it in the TPS for any clinically relevant discrepancy.

### 2.D The initial commissioning of the DIR tool for lung patient

It is difficult and time consuming to define corresponding landmarks for lung between CT and CBCT images. Instead, the landmarks on ten lung 4D‐CT cases from a public dataset (http://www.dir-lab.com) were utilized to evaluate the accuracy of the DIR tool on lung region.^40^, ^41^ Briefly, this dataset includes large numbers of evenly distributed landmark pairs (883.2 ± 507.7 per patient, 342–1561) identified on the end of inhale and exhale phases of the 4D‐CT. The DIRs were conducted without any parameter tuning of our tool and the output DVFs were evaluated with ground truth by Target Registration Error (TRE). The TRE was then compared to the published result and the interobserver variability provided with the dataset on its website (last checked on 2018/05).

4D‐CBCT has inferior quality and contains less structural details compared to 4D‐CT. For example, some vessel and bronchial bifurcations that are visible on CT may not be identifiable on CBCT. Therefore, the DIR algorithm may not always be able to find corresponding objects on the CBCT. Using the CT–CT landmarks validation, we intended to validate how well the DIR algorithm could model the respiration process and match the visible features inside the lung. In addition, CT and CBCT intensity have strong correlation^42^ and the LCC similarity metric employed in the final stage of the DIR is robust to this type of “modality transformation”.^43^ It also should be noted that the evaluation landmarks are defined on two extreme phases while the closest matching phases are chosen to minimize the difference before DIR in our application. Therefore, we believe the CT–CT validation results could serve as a surrogate for accuracy of the CT–CBCT DIR.

### 2.E Evaluation on clinical patients

Ten recently treated patients (six lungs SBRT, two head and neck, one breast, and one prostate) were retrospectively selected. Lung patients were chosen based on their large excursion variation. The other patients were selected for their large anatomic variation during treatment. This group of patients represented a sample of the most challenging cases in our clinic. The tumor position/excursion, dose prescription, and fractionation of all patients are list in Table 1. All the lung patients were treated with volumetric modulated arc radiotherapy (VMAT). During simulation and treatment delivery, patients were immobilized with an alpha cradle in the supine position, with arms above head. By default, the PTV_ITV_ is constructed as ITV plus 7 mm in the superior–inferior direction, and 5 mm in the other directions. Physicians may adjust the default margin based upon their experience. 4D‐CBCTs were taken for target alignment before treatment and for verification after treatment. The post‐treatment 4D‐CBCTs were used to reconstruct treatment dose for lung patients to include any intrafraction motion. The 4D Tx‐Dose was evaluated for the GTV coverage on the reference phase of the planning 4D‐CT. Head and neck (H&N) patient 1 had a single treatment plan; patient 2 had two plan modifications one at week two and another at week four. Both H&N patients were setup with face mask and daily CBCT guidance. The prostate patient is from an adaptive protocol of our institution, which defines a patient‐specific PTV (cl‐PTV) based on the convex hull of daily PTVs during the first week of treatment.^44^ The breast cancer patient was chosen for its relative large deformation during the treatment and no lymph nodes were treated. The precorrection CBCTs of the H&N, prostate, and breast patients were utilized to reconstruction their Tx‐doses by shifting the CBCT with the couch corrections recorded in the XVI database.

**Table 1:** Target coverage evaluated by cumulative treatment dose

Patient #	$\frac{Tx-plan}{\mathrm{plan}}\times 100$%			D_99_ (cGy)	Prescription (cGy × fractions)	Tumor position	Excursion (mm)	GTV (CC)
	D_99_ (%)	D_95_ (%)	D_90_ (%)					
Lung 1	96.5	97.4	98.1	6858.1	1200 × 5	RLL	6.0	2.34
Lung 2	94.7	96.4	97.4	5394.0	1200 × 4	LLL	6.1	1.57
Lung 3	93.6	93.3	93.5	5306.5	1200 × 4	RLL	5.7	8.94
Lung 4	100.1	102.1	102.6	5620.7	1200 × 4	RLL	7.1	6.18
Lung 5	94.4	96.7	97.5	5307.7	1200 × 4	RLL	13.2	3.69
Lung 6	83.7	84.6	86.3	5223.75	1000 × 5	RLL	7.8	0.85
Average	93.8	95.1	95.9				7.62	3.93
H&N 1	97.0	98.7	99.8	3779.7	180 × 22			
H&N 2	100.0	100.1	100.2	7150.9	200 × 35			
Breast	93.3	98.5	99.8	4203.1	200 × 25			
Prostate	101.30	102.20	103.40	7968.0	180 × 44			

## 3 RESULTS

**Figure 5:**
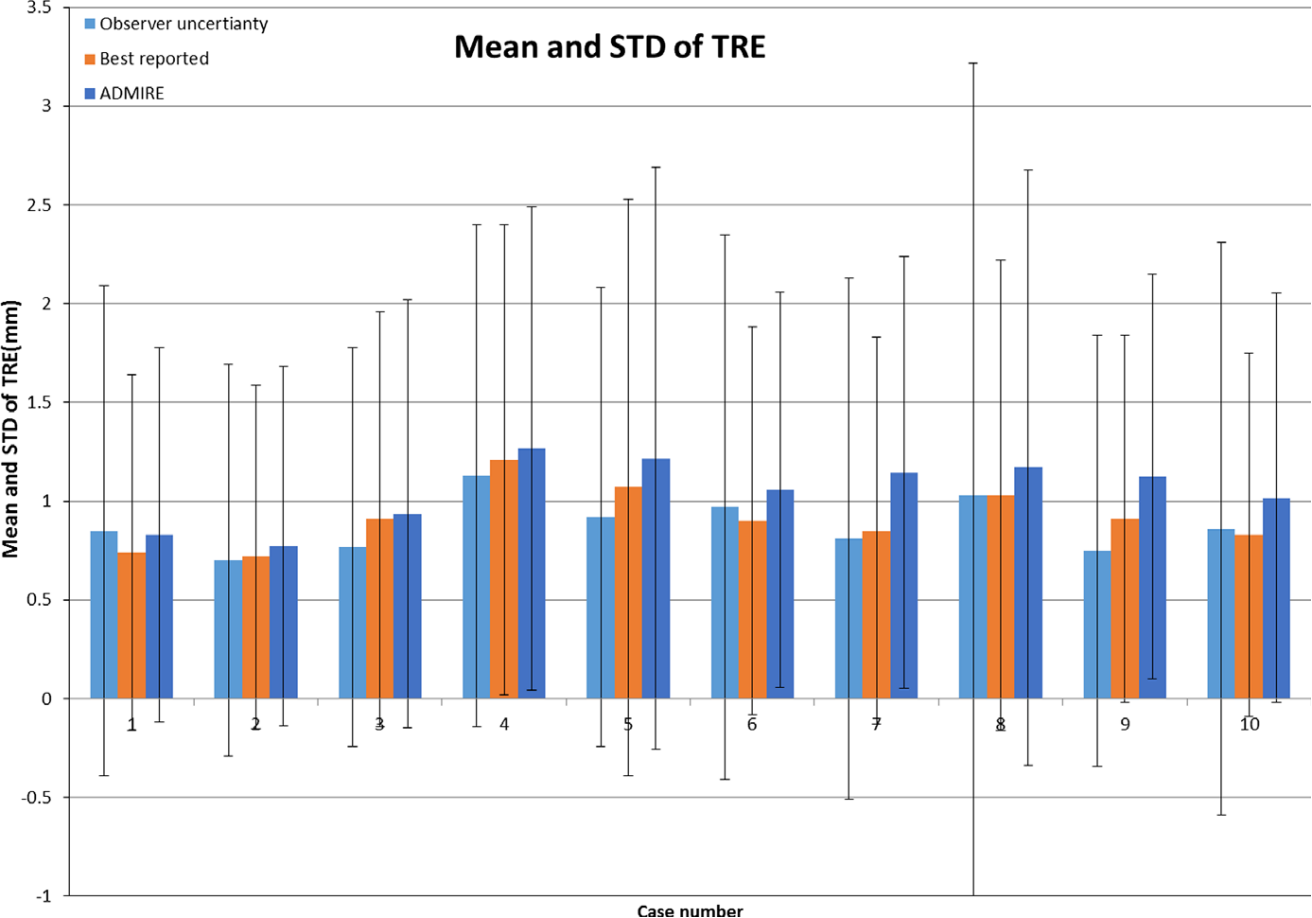
Mean and standard deviation of the landmarks TRE from DIR‐LAB ten cases.

Figure 5 shows the mean/STD of the TRE of our DIR tool per case, together with the best reported results and interobserver uncertainty from the DIR‐Lab website. The original landmark distance before DIR is 8.52 ± 5.57 (mean ± STD) mm. After DIR, the mean TRE of our DIR tool for all ten cases is 1.05 ± 1.13 mm, as compared with the interobserver uncertainty of 0.88 ± 1.31 mm and the best reported results 0.92 ± 1.05 mm. The best reported results were selected for each case and are from different algorithms. Our DIR tool demonstrated comparable accuracy to the best reported results and slightly lower uncertainty than expert observers. With GPU acceleration, the DIR of a typical lung CT–CBCT pair takes less than 1 min.

**Figure 6:**
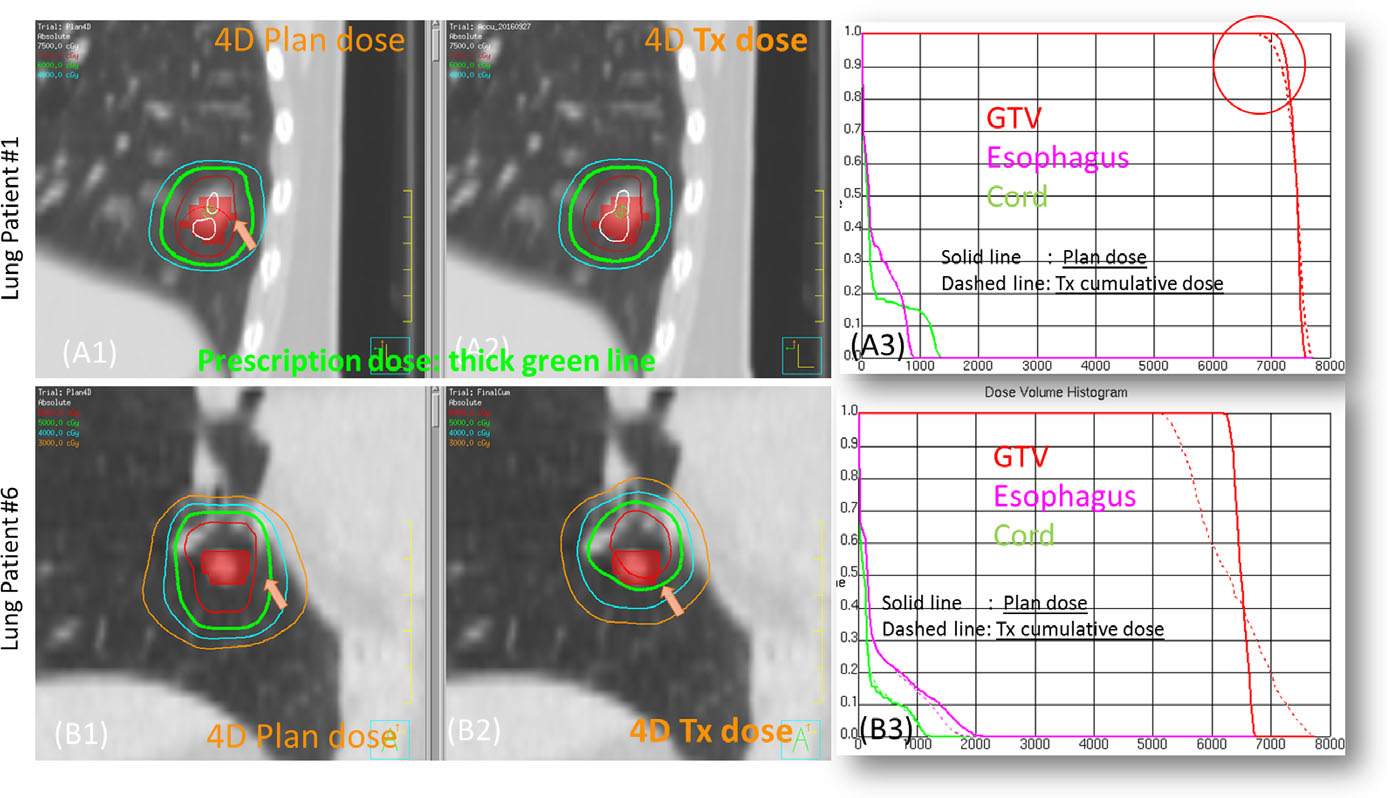
Example cases of lung SBRT patients: patient 1 (A1–A3) prescription 1200 × 5 cGy, patient 6 (B1–B3) prescription 1000 × 5 cGy.

The dose parameters representing target coverage for all ten clinical patients are listed in Table 1. It should be noted that the plan doses of lung patients were recalculated on ten phases and accumulated to the reference phase (4D plan‐dose). For all lung patients, the targets were well covered by the prescription dose as indicated by the GTV D_99_. Figure 6A(1–3) show the plan dose, final cumulative 4D Tx‐dose, and DVH for lung patient 1. Only a slight difference was observed in the high‐dose region inside the GTV (red color wash). The planned and final cumulative treatment doses of patient 6 are shown in Fig. 6B(1–3). For this patient, the daily setup was very challenging with a small tumor and vessel bifurcations nearby. Two out of five fractions were found to have inferior localization. The target was covered by the prescription dose as shown in the final cumulative doses, but much different than the planned one (Fig. 6B3).

**Figure 7:**
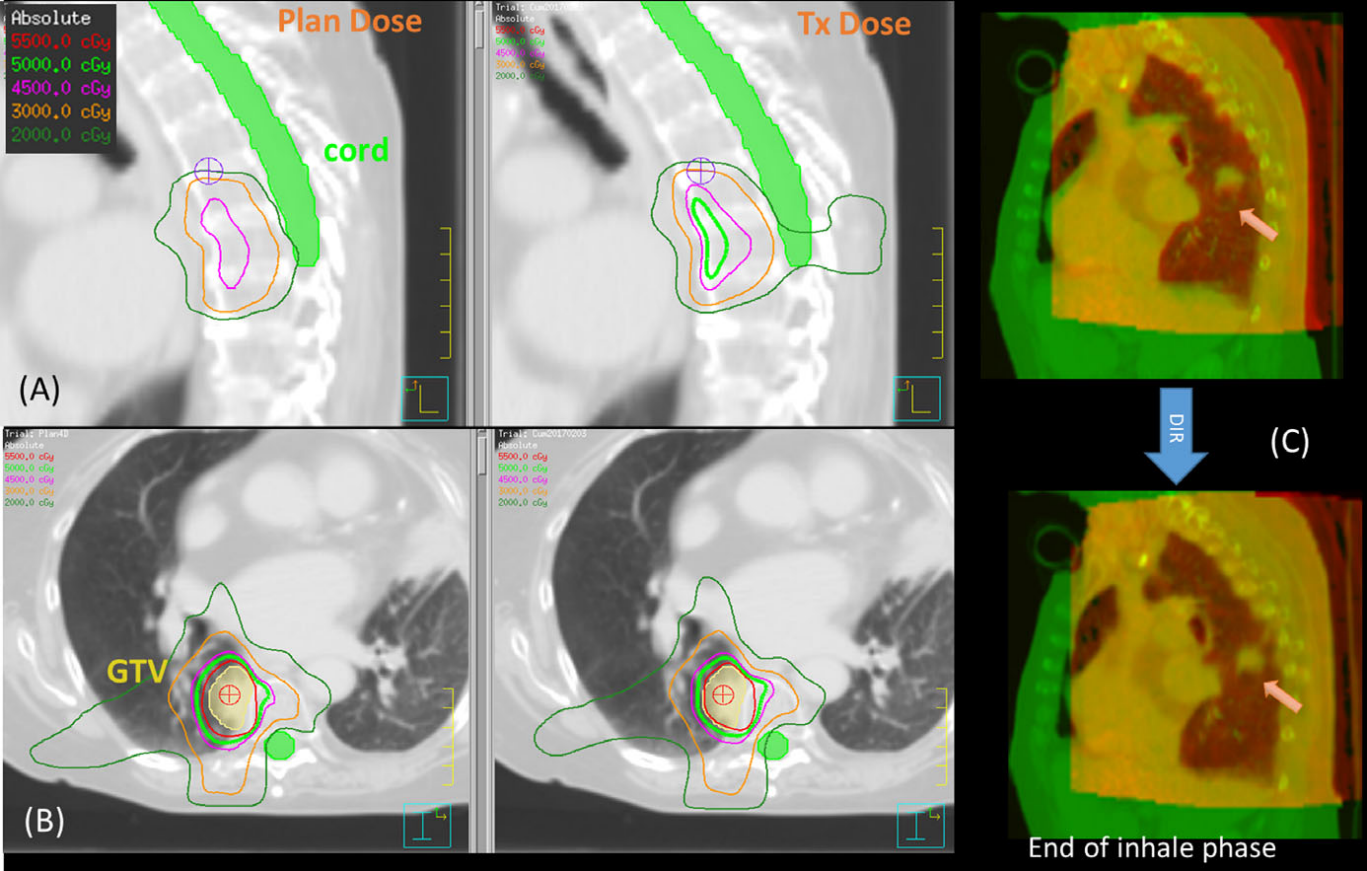
Inspection of plan dose and final treatment dose of lung patient 3.

The variations of clinically relevant dose parameters of OAR are listed in Table 2 for all lung patients. Variations up to 15% were observed for all OAR types in terms of maximum dose, which is very sensitive to organ geometrical changes. The sagittal and axial views of the dose distribution of lung patient 3 are shown in Figs. 7(A) and 7(B), respectively. The cord was spared quite well on the plan dose as shown by the 20 Gy Isodose line. On the final Tx‐dose, however, the cord got a higher dose as indicated by the invasion of the 20 Gy Isodose line into the cord. The fusion images of CT/CBCT before and after DIR on the end of inhale phase are shown in Fig. 7(C), with the arrow pointing to the tumor.

**Table 2:** The clinical relevant OAR dose parameters evaluated by cumulative treatment dose

Patient #	$Maxium\;dose:\frac{Tx-plan}{\mathrm{plan}}\times 100$%				Lung V20 plan (%)	Lung V20 Tx‐dose (%)
	Spinal cord (%)	Esophagus (%)	Aorta (%)	Heart (%)		
Lung 1	−3.6	9.1	−6.8	2.9	5	5
Lung 2	−6.6	3.6	0.1	0.7	5	5
Lung 3	15.7	13.1	4.7	−13.0	5	4
Lung 4	−2.9	−13.5	−15.6	−3.6	5	5
Lung 5	6.6	−2.2	5.9	3.0	3	3
Lung 6	−1.1	−7.9	−3.6	−17.7	3	2

**Figure 8:**
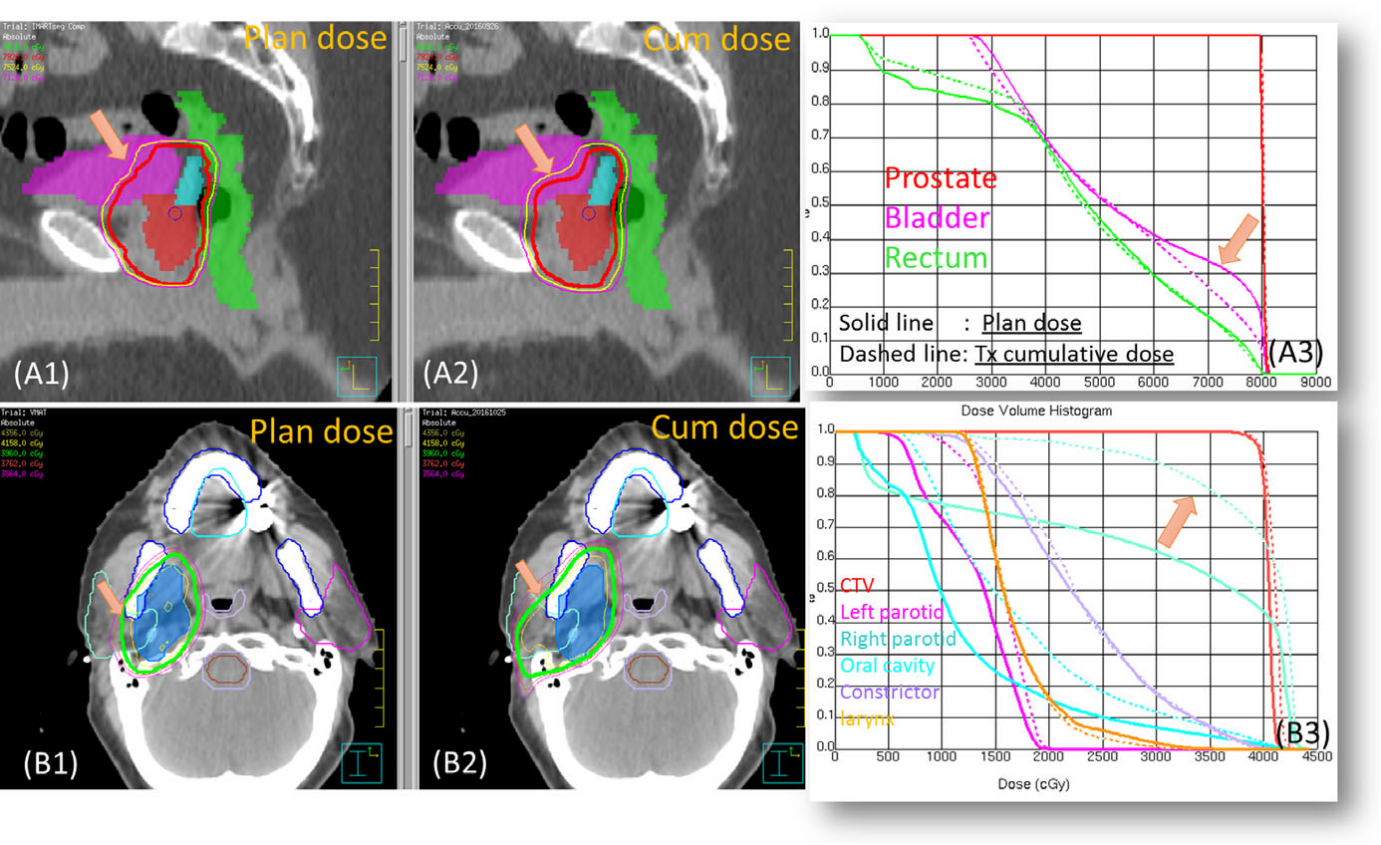
Example cases: prostate and head and neck patient 2.

The planned and final cumulative dose are shown in Fig. 8A(1–3) for the prostate patient and Fig. 8B(1–3) for H&N patient 1. While target coverage was maintained quite well, the dose to the ipsilateral parotid of H&N patient 1 became much higher due to the weight loss and parotid shrinkage. The lateral distance measured between the parotids center decreased by 1.39 cm in the last fraction, which means the parotid moved further into the high‐dose region. The volumes of the ipsilateral and contralateral parotid decrease by 48.8% and 36.5% respectively, as measured on the CBCT of the last fraction. Slightly lower bladder dose was observed for the prostate patient, which could be caused by the smaller bladder volume on the simulation CT (bladder volume: $\frac{plan\;CT}{CBCT\;average}=76\%$ . The target D_99_ of the breast patient, decreased by 6.7% compared to the planned dose.

## 4 DISCUSSION

We have demonstrated the effectiveness of an in‐house developed clinical treatment dose monitoring system on various treatment sites, with a special focus on lung SBRT patients. Using our novel phase‐matching DIR scheme between 4D‐CT and 4D‐CBCT, the reconstructed 4D treatment dose incorporates the variation of both tumor motion and surrounding density distribution, as manifested on 4D‐CBCT. By utilizing the functionalities of our existing clinical software and streamlining the workflow, this system has a flat learning curve and adds minimum extra workload for clinicians.

In the current implementation, the treatment doses are calculated overnight because the CBCT images cannot be directly accessed from our clinical CBCT imaging system due to practical reasons. Instead, the system retrieves CBCT's from a daily backup residing on a network disk drive. This limits the immediate availability of treatment dose for online evaluation. However, online treatment dose evaluation should be feasible if the CBCT data becomes accessible in real time. The DIR of single a CBCT/CT image pairs takes less than 1 min and the dose calculation, via the TPS (Pinnacle), takes another few minutes. The dose calculation time could be further reduced by employing a faster GPU dose engine like RayStation (RaySearch Laboratories AB, Stockholm, Sweden).

Another challenge of CBCT‐based Tx‐dose reconstruction is the limited FOV coverage. With the medium and large FOV available (covering 41 and 50 cm in diameter respectively), the Elekta CBCT can cover the whole treatment region for most H&N and prostate patients. The patching of CT outside the FOV for these patients should have minimum effect on Tx‐dose calculation. For lung patient, the ipsilateral lung is usually covered in whole. For a full arc VMAT delivery, up to one‐third of volume could be missing from CBCT FOV depending on target position [Fig. 2.B1]. In the current implementation, we patched the missing part with the corresponding 4D‐CT intensities of the matching phase. This could potentially lead to additional uncertainty because we are assuming the region outside the CBCT FOV is exactly the same as the plan CT on every fraction.

Patient‐specific HU tables have been proposed for dose calculation on 3D CBCT with acceptable accuracy for lung cancer patient.^23^ The HU/density table is generated by manually selecting the homogeneous areas of different tissues on both the CT and the CBCT, which is time consuming and error prone. And the HU variation and potential motion artifact of the CBCT could have more significant impact on dose calculation for lung regions with very low electron density. By utilizing 4D‐CBCT with the phase‐matching DIR scheme, the 4D treatment dose reconstruction should be a more suitable methodology for lung cancer patients, especially for SBRT delivery.

The newly published TG 132 recommends both quantitative and qualitative validation of the DIR tool for advanced clinical application like dose warping.^29^ The QA of the DIR algorithm, which is the under‐the‐hood key technique of this system, was done in two steps. The first step is the initial quantitative commission with a publicly available lung landmark data set and expert‐delineated contours. The second step is the daily patient‐specific qualitative assessment by fusion image and propagated contour. Currently, we set empirical thresholds for the volume change of target and solid critical organ (>10%) and for the translation of target center of mass (>3 mm). Any variation over the preset threshold will trigger notification to user. The user will inspect the autopropagated contour on the CBCTs as well as the fusion images to determine the DIR quality.

The frequency of human intervention to correct the propagated contours on the CBCTs depends on treatment sites. For H&N and lung patient, most fractions do not need user intervention if there is no significant artifact on CBCT. Prostate patient require the most frequent intervention due to the variation of bladder and rectum filling. Based on a survey of our dosimetrists who performed the editing of autogenerated contours (prostate, bladder, rectum, seminal vesicle), 10–40% of fractions needed editing of varying degrees, and the frequency is patient specific. In addition, a retrospective study could be conducted to identify certain patient characteristics that are associated with greater treatment dose deviation. For example, small centrally located lung tumor could be more susceptible to setup variation. Accordingly, only patients with high risk of dose variation are added to the tracking system to relieve workload.

Although the implementation of this system was based on our single institution's configuration, the system's modular architecture, generic XML file‐based treatment representation and well‐defined functional interfaces make the future extensions quite straightforward. For example, to take advantage of faster GPU based dose calculation, we plan to add RayStation as an optional dose reconstruction tool. This can be achieved by adding a new script generating function for the RayStation dose calculation/exportation in the TDR module without interfering with any existing functions. Future development is also planned for adding the CBCT‐based treatment dose monitoring for the spots‐scanning proton therapy, which suffers more substantial impact than photon treatment from anatomical variation with its sharp distal falloff.^24^, ^45^

## 5 CONCLUSION

The feasibility and effectiveness of a treatment dose verification system were demonstrated on various patient sites. A novel phase‐matching DIR between 4D‐CT and CBCT has been developed to reconstruct 4D treatment dose for lung SBRT treatment. This system can serve as an infrastructure for routine treatment quality monitoring as well as for outcome modeling based on true delivered dose.

## CONFLICT OF INTEREST

The authors declare no conflicts of interest.
